# Characterization of AISI 304 stainless steel based on laser cutting process optimization

**DOI:** 10.1038/s41598-025-24932-6

**Published:** 2025-11-20

**Authors:** A. S. Ali, Tamer Abdelmonem, Ramadan Elsoeudy, M. Abdel Mohsen, N. Elzayady

**Affiliations:** 1 Mechanical Engineering Department, Faculty of Engineering, Suez Canal University, Suez, 41522 Egypt; 2 Mechanical Engineering Department, Higher Technological Institute10th of Ramadan City (HTI), Ramadan City, 44629 Egypt; 3 Mechanical Engineering Department Faculty of Engineering, Ain Shams University, Cairo, 11566 Egypt; 4 Mechanical and Aerospace Engineering Department, Institute of Aviation Engineering and Technology, Giza, 12815 Egypt

**Keywords:** Fiber laser cutting, Surface roughness, Kerf geometry, Laser focus position, Microhardness, Mechanical engineering, Structural materials, Characterization and analytical techniques

## Abstract

This study investigates the impact of cutting speed and laser focus position on surface roughness, kerf geometry, and microhardness during fiber laser cutting of 4 mm-thick AISI 304 stainless steel sheet. A series of experimental trials were conducted using fiber laser cutting with cutting speeds ranging from 1.5 to 3.5 m/min and focus settings between 30 and 50%, at fixed power of 2000 W and gas pressure equals 14 bar. Surface roughness (Ra) was found to decrease with increasing cutting speed; the highest Ra value recorded was 5.4 µm at 1.5 m/min and 40% focus, while the lowest value was approximately 4.0 µm at 3.5 m/min and 50% focus. Kerf width similarly decreased with increasing speed and optimal focus, narrowing from 185 µm at 1.5 m/min and 30% focus to as low as 138 µm at 3.5 m/min and 50% focus. Microhardness varied non-linearly, reaching a peak of 270 HV at 2.0 m/min and 30% focus, indicating favorable thermal gradients. SEM, EDS, and XRD analysis validated the outcomes indicative of a balanced performance across all evaluated parameters. These findings guide laser cutting optimization for improved precision and mechanical performance in stainless steel.

## Introduction

Laser cutting is a high-precision and low-waste solution for many manufacturing processes. It is a major area of global research due to its many benefits. It boasts fast cutting speeds and produces very little waste. Unlike traditional methods, it doesn’t cause surface damage or leftover stress. Besides, it’s perfect for hard-to-reach or highly radioactive spots. Since it’s a non-contact method, laser cutting tools also have an extended lifespan. This technology is highly versatile, making it ideal for complex and precise tasks^1,2^. Other researchers defined it as a non-contact thermal process that is widely utilized in modern industries due to its low cost and ease of use^3–8^. The commonly used Laser processing nowadays are laser cutting^9,10^, cladding^11^, punching^12,13^, marking^14^, polishing^15^, cleaning^16^ and surface refinement^17,18^. Laser cutting, is designated as a thermal-based separation process that provides an efficient method for the fabrication of sheet metal products. In comparison with conventional techniques such as punching, it offers superior flexibility with respect to material applicability, utilization efficiency, geometric adaptability, and ease of integration into industrial manufacturing systems^19^. The capability of laser cutting to generate intricate profiles with high dimensional accuracy and minimal material distortion has positioned it as a leading technique in various manufacturing sectors including aerospace, automotive, electronics, and medical equipment production. Among commonly processed materials, AISI 304 stainless steel of varying thickness. Sheets with 4 mm thickness produced from this material are extensively utilized across industries. AISI 304 stainless steel is well known for its lower cost than AISI 316 while providing sufficient corrosion resistance for typical applications, mechanical durability, and ease of fabrication. In the food and beverage sector, this material ensures hygienic performance, while in chemical and pharmaceutical applications, it provides both chemical resistance and reliable weldability under pressure. For construction and architectural applications, it combines aesthetic appearance with external corrosion resistance and structural rigidity^20,21^ In the automotive and transport industries, it withstands elevated temperatures and chloride exposure while maintaining an optimal strength to weight ratio. Its resistance to marine corrosion ensures integrity in splash-zone environments, and in pressure vessels and heat exchangers it delivers both pressure tolerance and durability under thermal cycling. Furthermore, in medical and sanitary equipment, its non-toxic and sterilizable nature ensures reliability under repeated use^22–26^.

Essentially, laser cutting operates with a focused, high-energy laser beam to generate localized heating of the material, leading to melting. The molten material is then expelled by a high-pressure assist gas or other removal mechanism, resulting in a clean-cut edge^24,27,28^. Unlike mechanical cutting, laser cutting eliminates tool wear, vibration, and excessive mechanical forces, enabling the processing of hard, brittle, or thin materials with superior quality. The process is influenced by numerous factors including the material’s thermal characteristics such as thermal conductivity, absorptivity. Also, laser features like wavelength and power influence the cutting process. Additionally, operational parameters such as cutting feed rate, focal position, and gas type affect the process. In fiber laser cutting, the choice of assist gas significantly impacts performance, with air, carbon dioxide, argon, nitrogen, and oxygen being the main choices. Nitrogen is widely preferred in both research and industry due to its inert nature, which prevents oxidation and discoloration, ensuring high surface quality for materials like stainless steel, coated metals, and aluminum. High-pressure nitrogen flow also allows for smoother, burr-free edges that limit post-processing and is effective on a wide range of materials and thicknesses. Compared to argon, nitrogen is more cost-effective, while oxygen, beneficial in increasing the material removal rate through exothermic reactions, affects surface quality and is therefore preferred when cutting speed is prioritized over edge integrity.^1,29^. There are several modes of laser cutting depending on the interaction mechanism: fusion cutting (melting with inert gas), flame cutting (oxidation with active gas), vaporization cutting, and chemical degradation cutting^30,31^. Each of these modes impacts the kerf width, heat-affected zone (HAZ), and surface quality differently. For metals processing, CO₂ lasers and fiber lasers are the most used. CO₂ lasers are preferred for thick-section materials, particularly steels, due to their greater penetration depth, while fiber lasers are more effective for reflective materials like copper, and aluminum owing to their higher absorption efficiency at shorter wavelengths^32,33^.

Feed rate is one of the most significant factors impacting surface roughness in laser machining. The interaction time between the material and the beam decreases by increasing the feed rate (cutting speed) , generally leading to a smoother surface finish. Also, the microhardness of cut surfaces is impacted by the applied cutting speed, primarily due to the thermal cycles imposed on the material during machining. Increased cutting speed results in smaller heat-affected zone (HAZ) and thus lower thermal softening, preserving the material’s original hardness or leading to hardening due to rapid cooling^1,30^^34–36^

Ozaki et al. (2012) studied austenitic stainless-steel (SUS304) and demonstrated that increasing the feed rate from 10 mm/s to 100 mm/s (at a constant 2.0 kW laser power) reduced both the area affected by heat, and kerf width and, resulting in more uniform hardness across the cut edge. The kerf width decreased from 0.56 mm to 0.25 mm, and the heat input dropped below 20 J/mm, leading to minimized thermal degradation^32,37^.

In a study by Jarosz et al. (2016), laser cutting of AISI 316L stainless steel showed that increasing the cutting speed from 1.84 mm/s to 16.5 mm/s significantly reduced roughness of the cut surface. At the lowest speed (1.84 mm/s), the surface roughness parameter Ra at the cut exit was 5.14 µm, while at the highest speed (16.5 mm/s), Ra was 7.53 µm, indicating that excessively low speeds increase roughness due to heat accumulation and dross formation. On average, the middle zone of cuts produced at 9.17 mm/s had Ra ≈ 2.62 µm, which was lower than that at 1.84 mm/s, suggesting an optimal range for balancing quality and productivity^30^.

Similarly, the study reported that critical cutting speed (Vc) values increase with laser power, enabling higher productivity without compromising hardness. For example, at 2.0 kW, 100 mm/s was the pivotal speed while at 1.0 kW it was only 50 mm/s. This indicates that higher cutting speeds, coupled with adequate power, can achieve full cuts with limited hardness variation, as the reduced HAZ thickness prevents microstructural changes that typically cause hardness loss^32^.

Additionally, Genna et al. (2020) in the process of AISI 304 and AlMg3 laser cutting employing CO₂ assist gas, observed that the surface roughness (Ra) decreased with higher cutting speeds across all materials tested. This trend is attributed to reduced thermal energy per unit area, minimizing molten metal deposition and drag line formation along the kerf surface. For example, roughness values below 2.5 µm Ra were achieved at high speeds (~ 8.9 m/min), while lower speeds (~ 2.3 m/min) resulted in Ra values above 5 µm, depending on gas pressure and focus position^38^. Generally, increasing cutting speed leads to narrower kerfs due to reduced heat input per unit length, which minimizes material melting and ejection^38^.

Mahrle et al. (2021) conducted factorial experiments on 10 mm thick AISI 304 using a 4-kW fiber laser and observed that higher cutting speeds resulted in narrower kerf widths and better edge quality. The kerf geometry was affected more by intrinsic factors such as the internal flow field and molten material behavior than by the cutting gas parameters themselves^39^.

Xu et al. (2024) evaluated the multi-layer cutting of 304 stainless steel plates and found a clear correlation between cutting speed and kerf width. At 8000 W laser power, reducing feed rate from 900 mm/min to 400 mm/min increased the kerf width in the fourth layer from 1.68 mm to 2.85 mm, indicating that excessive heat input at low speeds result in larger kerf dimensions due to more extensive melting^34^.

Similarly, in another study, kerf width was minimized at a critical cutting speed of 50 mm/s for SUS304 using assist-gas-free laser cutting. Above or below this threshold, kerf width increased due to inefficient melting or ejection of the molten pool^32^.

Focus point position, which defines the distance between the workpiece surface, and the laser’s focal plane, is an essential factor in laser cutting that directly influences surface roughness through changes in energy density and interaction depth. When the focus is optimally positioned within or just below the material surface, it facilitates concentrated energy delivery, yielding cleaner cuts and reduced roughness. Conversely, too much positive or negative defocus leads to beam dispersion, inefficient melting, and striation formation on the cut edge^40^. Focal point positioning also impacts the hardness of the HAZ and the cut edge due to differences in thermal load and cooling rate. A tightly focused beam increases local energy density, leading to deeper penetration and more thermal input, which can soften or harden the material depending on cooling conditions and material type.

Wardhana et al. (2019) in the process of 316L laser cutting, concluded that optimal surface quality was obtained when the focal point was adjusted near the surface, minimizing energy losses and striation marks. The lowest surface roughness values were recorded at focal point positions around 0 mm ± 1, with Ra ≈ 2 µm, compared to higher Ra values (> 3 µm) at more defocused settings^36^. Also, cutting 316L with varied parameters showed that deeper penetration from optimal focal adjustment increased the localized hardness, especially when paired with high gas pressure. The surface hardness near the cut was observed to be higher than the base metal, suggesting rapid self-quenching from the laser-induced melt layer contributed to hardness increases in those zones^36^

In another study by Zubko et al. (2020) observed changes in the microstructure and phase composition of material waste resulting from laser cutting of AISI 304. Though the study did not manipulate focal point directly, it highlighted that beam positioning affects phase transitions and the formation of hard oxides (e.g., Fe₃O₄ and α-Fe) near the cut edge. These structural transformations are known to influence surface hardness, especially under nitrogen shielding conditions that prevent oxidation^41^.

A study by Buj-Corral et al. (2021), further confirmed that focal distance during laser cutting has a significant impact on surface roughness of thin stainless-steel plates. They conducted experiments using a full factorial design and found that focal position interacted strongly with pulse width and frequency. Surface roughness (Ra) varied between 1.89 µm and 3.86 µm, with the smoothest surfaces achieved at focal distances close to the material surface and at high speed and frequency^41^.

Mahrle et al. (2021) demonstrated that focal plane position has a strong influence on kerf geometry in laser cutting of AISI 304 stainless steel. Their factorial analysis showed that positioning the focus within the material (i.e., negative focus values) enabled better penetration and narrower kerf formation. The optimal focus point helped maintain kerf width around 0.4 mm, while poor focus settings resulted in uneven kerfs and dross formation^39^.

Xu et al. (2024) studied the effect of defocus amount, ranging from − 10 mm to − 34 mm, in high-power fiber laser cutting of four-layer grid plates. At a defocus setting of − 14/ − 30 mm, kerf width in the fourth layer varied between 1.61 mm and 2.85 mm, depending on other parameters such as cutting speed and gas pressure. Larger defocus values (more negative) caused kerf widening due to reduced laser intensity at the cutting zone, especially in deeper layers^34^. Moreover, in nuclear fuel rod disintegration simulations, optimal defocus values ensured better laser beam penetration through upper layers, maintaining consistent kerf dimensions even with varying auxiliary gas effectiveness. Excessive defocusing led to inefficient cutting and inconsistent kerf shapes, particularly in multi-layer assemblies^34^.

AISI 304 stainless-steel finds broad application in industrial fields due to its excellent combination of formability, corrosion resistance, and mechanical strength, particularly in 4 mm thickness for laser cutting and fabrication as mentioned by Zatkalíková et al. in 2022 and by Mroczkowska et al. in 2021^42,43^.. Its strength and ductility support complex shapes without compromising integrity^44,45^. Advanced processes such as laser cutting, welding, and femtosecond texturing further enhance its performance in structural and hygienic applications across sectors like construction and food processing industries^46^.

Despite the proliferation of studies investigating the influence of laser cutting parameters on metallic materials, a notable research gap persists regarding the behavior and optimization of widely used industrial alloys such as AISI 304 stainless steel. This austenitic stainless-steel grade, distinguished by its excellent corrosion resistance, formability, and mechanical strength, is extensively applied in sectors ranging from medical equipment and food processing to structural components and energy systems. However, the laser machining characteristics of AISI 304 including its thermal conductivity, surface roughness, and microstructural response to high-intensity beams remain underexplored in the context of parameter optimization for both performance and sustainability.

More critically, current literature often emphasizes empirical trends without translating findings into actionable operating envelopes that balance cut quality, material integrity, and energy efficiency. For instance, while higher cutting speeds reduce energy consumption, they may induce thermal gradients that degrade edge quality or promote HAZ microcracking. The lack of a standardized framework for defining and validating optimized parameters such as laser power, assist gas pressure, and cutting speed for different laser types (fiber, CO₂, or diode lasers) limits the scalability of sustainable machining practices across industries.

Addressing this gap would not only enhance industrial competitiveness but also align with broader environmental sustainability goals by reducing energy input, material waste, and post-processing requirements. Therefore, there is an urgent need for comprehensive experimental and numerical studies that evaluate the interactions between laser parameters and the thermo-mechanical response of various materals. These studies should aim to develop optimization frameworks that minimize energy consumption while maintaining cut precision and material performance, thus contributing to the next generation of sustainable manufacturing technologies.

The current research aims to optimize fiber laser cutting of AISI 304 stainless steel by analyzing the effects of cutting speed and focus position on surface roughness, kerf geometry, and microhardness AISI 304 was selected as the workpiece material due to its cost-effectiveness compared with AISI 316, (30% lower cost than AISI 316) making it a practical choice for applications where moderate corrosion resistance is sufficient. Under nitrogen-assisted fiber laser cutting, both alloys exhibited high edge quality with minimal post-processing requirements, due to the absence of oxidation and the advantage of avoiding costly gases such as argon, which is approximately 300% more expensive than nitrogen. This study investigates how these parameters influence cutting quality and mechanical properties, using SEM analysis to assess surface morphology across cutting paths to identify optimal settings for efficient industrial applications. Also measuring microhardness and surface roughness to assess mechanical properties effects, then finally measuring the cut geometry to assess the cutting quality.

## Material and Method

### Material

The material used in the current study is AISI 304 stainless steel with a thickness of 4 mm, its chemical composition shown in Table 1 was obtained through energy-dispersive X-ray spectroscopy (EDS) analysis performed during sample preparation using an Elementar Vario Lab elemental analyzer at the Central Metallurgical Research and Development Institute (CMRDI), Tebbin, Helwan. AISI 304 stainless steel is a prevalent and standardized substance in the metal sheet industry. It comprises Ni and Cr, which facilitate the absorption of laser energy while exhibiting delayed heat dissipation. Consequently, it yields favorable outcomes in laser cutting. The hardness of 304 St. St. the utilized in this study is 180 HV. Square samples measuring 20 mm $\times$ 20 mm were cut-off.

**Table 1:** Chemical Composition of AISI 304.

Weight %								
Element	C	Si	Mn	P	S	Cu	Cr	Ni
Avg	0.022%	0.384%	1.104%	0.017%	0.012%	0.218%	18.35%	7.77%
Element	V	Ti	Co	W	Sn	N	Mo	Fe
Avg	0.101%	0.009%	0.17%	0.004%	0.013%	0.435%	0.209%	71.18%

### Method

**Fig. 1:**
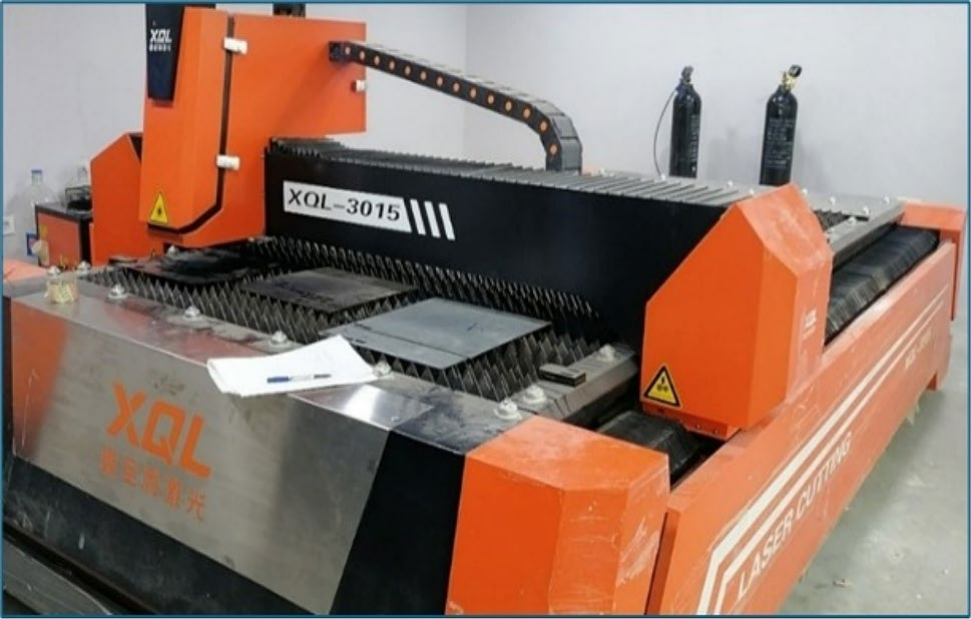
Photographic view of laser cutting machine model XQL-3015.

XQL-3015 is an industrial-grade fiber laser cutting machine featuring a 2000W IPG laser source, optimized for high-precision cutting of metals like stainless steel, carbon steel, and aluminum. Figure 1 shows the picture of this machine, it includes a 1500 $\times$ 3000 mm workspace, an auto-focus WSX cutting head, and a sophisticated CYPCUT control system. Schneider servo motors and Motovario reducers provide stability and precision (< 0.1 mm/m), while a dual-drive gearbox facilitates speeds of up to 120 m/min. The cutting parameters that are typically not altered for each operation were maintained constant. Their values are displayed in Table 2.

**Table 2:** Specifications of XQL-3015 Laser Cutting Machine.

Model	XQL-3015
Laser Power	2000 W
Control System	CYPCUT 2000 (Shanghai Bochu)
Max Running Speed	120 m/min
Laser Type	Fiber Laser
Positioning Accuracy	< 0.1 mm/m

**Fig. 2:**
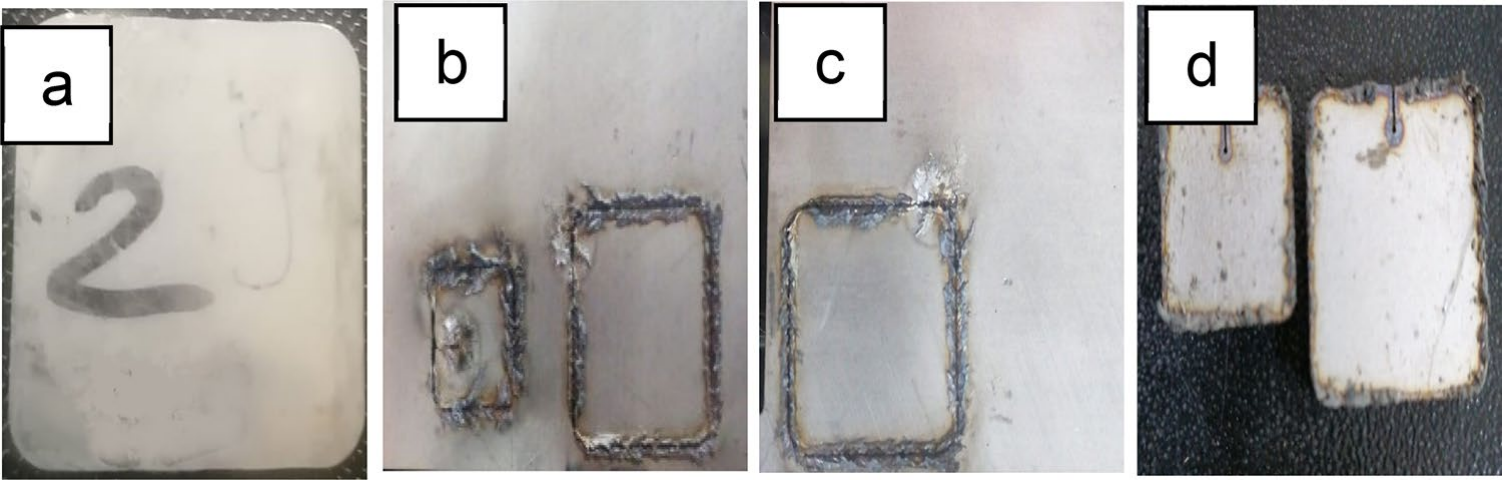
**a** Experiment sound specimen, **b** Preliminary experiment specimen at 1800 W, **c** Preliminary experiment specimen at 8 bar gas pressure, and **d** Preliminary experiment specimen at 1800 W at 10 bars gas pressure.

**Fig. 3:**
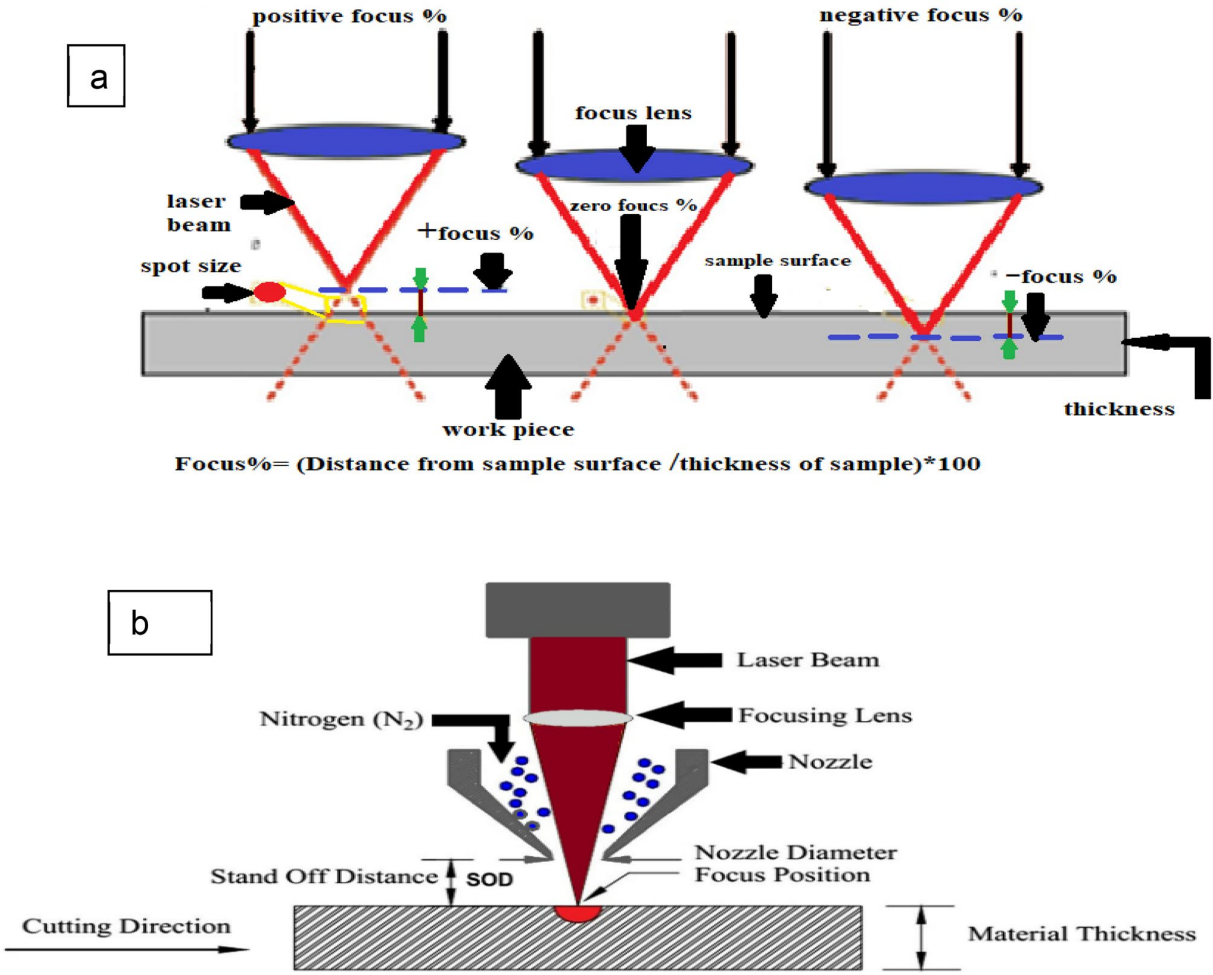
**a** Focus position, **b** Schematic of laser cutting with indicated parameters^1^.

A series of laser cutting process parameters used in the experimental design with different ranges. The ranges of parameters used in the preliminary experiments are as follows: laser power of 1600, 1800, and 2000 W,W; gas pressure of 8, 10, 12, and 14 bar; stand-off distance (SOD) of 0.3, 0.5, 0.7, and 1 mm; cutting speed of 1, 1.5, 1.7, 2, 2.2, 2.5, 2.8, 3, 3.5, and 4.5 m/min; and laser focus position of 30%, 35%, 40, 45%, 50%. Figure 2 presents specimens with different specifications, including both sound and defective ones. Based on the results of the preliminary experiments, 15 different cutting process combinations were recommended in the current investigation. Table 3 displays the parameters range of these combinations. As presented in the Table 3, the selected experimental ranges for cutting speed (1.5–3.5 m/min with increments of 0.5 m/min.) and laser focus position (30–50% with increments of 10%.) were selected based on two considerations: (1) they fall within the typical industrial workload for fiber laser cutting of 4 mm thick stainless steel sheet using a 2000 W laser source and high assist gas pressure (14 bar), and (2) they aimed to explore subtle process variations that might affect cut quality and thermal response, particularly when transitioning between low and high power densities. These defined limits were guided by preliminary experiments and supplier guidelines, shown in Fig. 2, ensuring their suitability for both academic research and industrial practice. Therefore, two key variables were defined: feed rate (m/min) and negative focus (%) shown in Fig. 3a, and the laser cutting process schematic, shown in Fig. 3b, to evaluate their impact on cut quality at 0.3 mm SOD. The laser power (100%) and gas pressure (14 bar) were kept constant throughout the experiments to isolate the effects of other parameters.

**Table 3:** Setting parameters for laser cutting trials.

No	Feed rate (m/min.)	Focus (%)	No	Feed rate (m/min.)	Focus (%)	No	Feed rate (m/min.) (m/min.)	Focus (%)
1	1.5	30	6	1.5	40	11	1.5	50
2	2	30	7	2	40	12	2	50
3	2.5	30	8	2.5	40	13	2.5	50
4	3	30	9	3	40	14	3	50
5	3.5	30	10	3.5	40	15	3.5	50

## Testing

### Surface roughness measurements

**Fig. 4:**
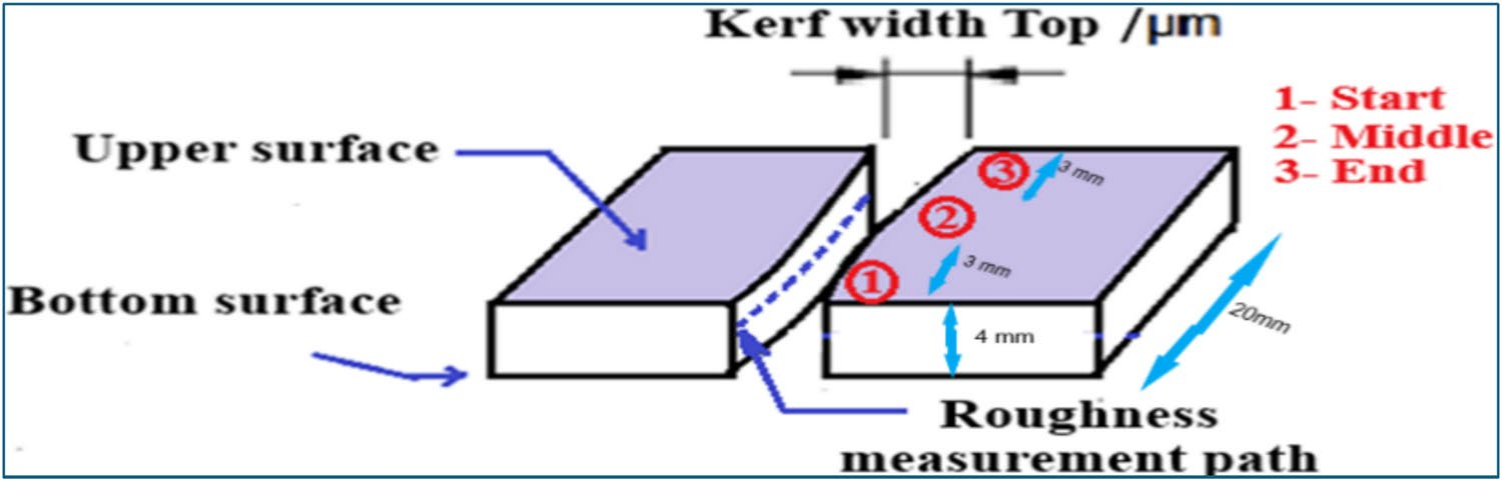
Measurement zones for kerf width and cut edge surface roughness.

A surface roughness tester is a precise tool employed for evaluating the roughness of a material’s surface by tracing its profile with a stylus (TR 200—SaluTron Messtechnik GmbH). Figure 4 shows measurement zones for surface roughness. Roughness values, a standard metric for assessing cut edge quality. These values were derived from profile measurements on both sides of the cut edge, expressed as Ra (arithmetical mean deviation of the evaluated profile) and Rz (average distance between the highest peak and lowest valley) at various vertical positions of the cut right-hand side. Figure 4 illustrates the measurement details. The cut kerf geometries are characterized by the edge. The kerf width is measured on the upper surface at the start, middle, and end of the cut using a Moticam 2300 digital microscope.

**Fig. 5:**
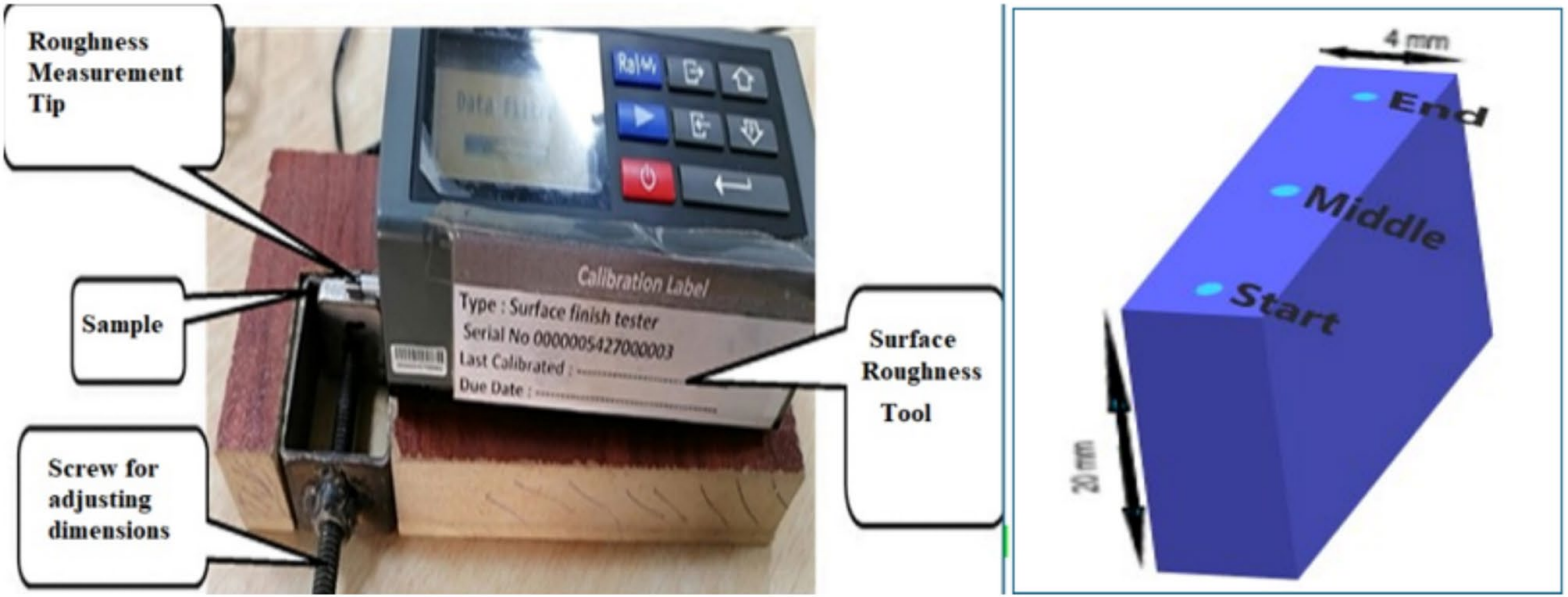
Support fixation for surface roughness measurement, microhardness sample spots positions.

Figure 5 shows a laser-cut edge profile used to measure microhardness on the surface at three positions (start, middle, and end) of the cut edge. Surface roughness was also measured twice and averaged along the middle of cutting path from start to end. These measurements statistically help to evaluate the cut quality and surface roughness, providing essential insights into laser cutting performance**.**

### Hardness measurements

The microhardness of the specimens is assessed using the FM-800 microhardness tester with a load of 100 gf and a dwell time of 10 s according to HV. Figure 5 shows microhardness specimen.

### Microstructure analysis

Microstructure samples were analyzed by laser scanning confocal microscopy (LSCM, VK—× 200, Keyence Ltd., Osaka, Japan) and a field emission scanning electron microscope (FESEM, Carl Zeiss Sigma AG, Oberkochen, Germany). Additionally, the cut samples were subjected to XRD evaluation using a BRUKER D2 PHASER (2nd Gen) instrument.

## Results and discussions

### Effect of cutting speed on surface roughness

**Fig. 6:**
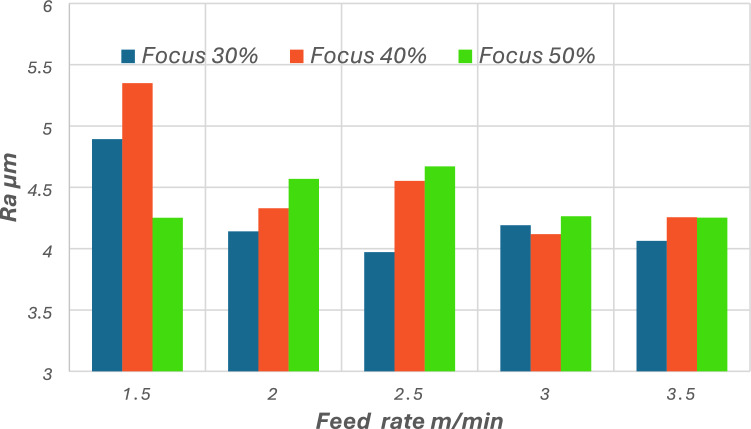
Surface roughness at different cutting speeds and focus positions.

Figure 6 demonstrates the correlation between feed rate (cutting speed) and surface roughness (Ra) at various laser focus settings (30%, 40%, and 50%). Surface roughness often increases with a decrease in cutting speed, irrespective of the focus %. At a minimal cutting speed of 1.5 m/min, the surface roughness peaks at roughly 5.4 µm at 40% focus, signifying a suboptimal surface finish attributable to extended laser-material interaction and possible thermal damage. Conversely, when the cutting speed is increased from 1.5 to 3.5 m/min, Ra values ​​decreased significantly^47^, with the lowest value being recorded at approximately 4.0 µm at 30% concentration as shown in Fig. 6. This indicates that increasing cutting speeds produce smoother cuts by reducing transmitted energy density, which reduces surface degradation. The effect of concentration variation is evident at most rates, with increasing the concentration from 30 to 40% typically increasing Ra, while increasing it to 50% typically deteriorates surface quality at moderate focus settings (30–40%), the laser beam is well-concentrated near or slightly below the surface of the material, ensuring high energy density at the cutting interface. This facilitates efficient material removal with minimal thermal damage or melt residue. However, increasing the focus position to 50% effectively shifts the focal point farther from the material surface, resulting in beam defocusing and a broader spot size. This reduces the intensity of the beam per unit area, leading to less efficient energy transfer, inconsistent melting, and potential re-solidification of partially melted material on the cut edges, which elevates surface roughness.

Moreover, the increase in beam divergence and associated energy density fluctuations at 50% focus contribute to instabilities in the melt flow and gas-assisted ejection, producing surface irregularities. These physical phenomena explain why surface quality deteriorates at higher focus percentages despite smoother performance at intermediate settings. At a cutting speed of 2.5 m/min, Ra is approximately 4.6 µm at 40% concentration, in contrast to 4.0 µm at 30% concentration. The interaction time between the material and the laser beam decreases, thereby reducing the total thermal energy input per unit area. This mitigates excessive melting and results in less molten metal re-deposition on the cut edge, which in turn reduces surface irregularities. Moreover, higher speeds minimize the formation of drag lines, which are typically caused by prolonged melt expulsion along the cut wall. These effects collectively explain why the surface roughness (Ra) decreased from 5.4 µm at 1.5 m/min to approximately 4.0 µm at 3.5 m/min, as observed in our experiments.

### Effect of cutting speed on kerf geometry

**Fig. 7:**
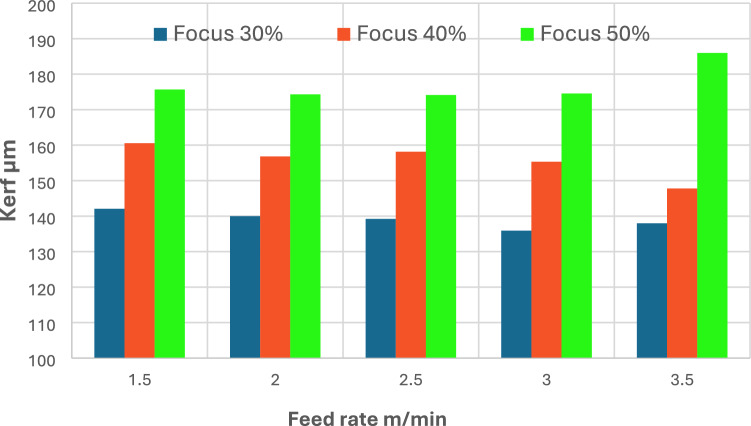
Kerf width variation under different feed rates and laser focus settings.

Figure 7 shows the variation in kerf width (μm) as a function of cutting speed (m/min) for three different laser concentration settings: 30%, 40%, and 50%. Kerf width is shown to decrease continuously with decreasing concentration at all cutting speeds, indicating that concentration has a major influence on cutting accuracy. At the highest cutting speed (3.5 m/min), kerf width reaches approximately 185 μm at 50% concentration, decreases to 148 μm at 40% concentration (25% improvement), and then narrows further to approximately 138 μm at 30% concentration (7% improvement). Similar patterns are observed at lower cutting speeds. For example, at 2.5 m/min, kerf widths are approximately 175 μm, 158 μm, and 138 μm at 50%, 40%, and 30% concentration levels, 15% and 10% improvements respectively. Interestingly, while cutting speed has a minor effect on the kerf, the focus ratio plays a more significant role in optimizing the laser cut width. The narrowest kerf values ​​are consistently achieved at 30% focus across all feed rates, indicating a more focused and efficient energy distribution, which reduces the material removal width. In contrast, higher focus ratios result in wider kerfs due to beam defocusing and energy dispersion. The decrease in surface roughness (Ra) at elevated cutting speeds and optimal focus (30–40%) is ascribed to reduced contact time, increased cooling rates, and diminished melt pools, which curtail dross production and striation markings. At reduced velocities or increased focus (e.g., 50%), extended thermal input and diminished energy density result in unstable melting dynamics, producing rougher surfaces and broader heat-affected zones. Likewise, the reduction in kerf width at diminished focus percentages (e.g., 30%) results from heightened beam concentration and energy density, facilitating effective and accurate material removal. Conversely, a defocused beam (more percentage) disperses energy, resulting in broader kerfs due to diminished localized heating and less efficient melt expulsion. These data demonstrate the significant impact of melt pool dynamics, energy distribution, and thermal gradients on the ultimate quality of the cut.

### Effect of cutting speed on microhardness

**Fig. 8:**
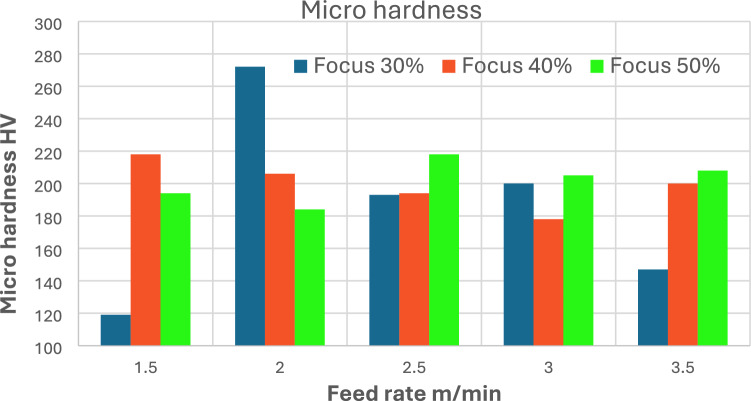
Influence of feed rate and laser focus on microhardness.

Figure 8 shows the microhardness (HV) variation of the laser-cut samples, affected by different cutting speeds and feed rates (30%, 40%, and 50%). The results demonstrate a nonlinear relationship between the cutting parameters and the resulting microhardness values ​​of the material. At the lowest feed rate of 1.5 m/min, microhardness is significantly higher at 40% focus, reaching approximately 220 HV, while it drops sharply to approximately 115 HV at 30% focus, indicating excessive thermal load leading to surface degradation due to the slower cutting speed. In contrast, at 30% focus and 2.0 m/min cutting speed, the highest hardness value is achieved amongst all experiments, approximately 270 HV, indicating an optimal balance between input power and cooling rate that promotes precise microstructural transformation, as subsequently demonstrated by scanning electron microscopy (SEM) analysis. As the cutting speed increases to 2.5 m/min, the microhardness decreases slightly, ranging between 190–220 VHV, with the 50% focus giving the peak value.

At higher cutting speed of 3.5 m/min, the microhardness continues to decrease slightly, stabilizing around 200, 210 HV for both 40% and 50% focus settings, respectively. At 30% focus, hardness performance is poor at 150 HV. This suggests that moderate feed rates with finer beam focus (50%) enhance overall hardness, likely due to faster cooling and improved microstructure, while cutting speeds that are too low or too high leads to thermal softening or uneven energy distribution. This behavior highlights the importance of optimizing cutting parameters for the industrial sector and how it can contribute to improved productivity with minimal waste.

### Metallography examination

#### Scanning electron microscope (SEM) evaluation of cut surface quality

**Fig. 9:**
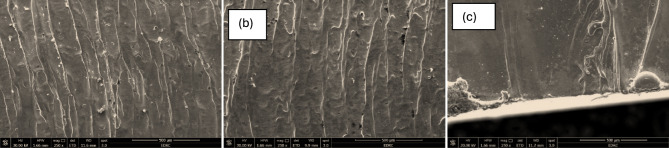
SEM images showing the initial cutting surface morphology of AISI 304 stainless steel under different laser cutting speeds: **a** Sample 1 at 1.5 m/min, **b** Sample 4 at 3.0 m/min, and **c** Sample 15 at 3.5 m/min.

The SEM micrographs illustrate the effect of laser cutting speed on the surface quality of AISI 304 stainless steel at the beginning of cutting providing clear insight into how melting dynamics, surface roughness, and defect formation are influenced by cutting speed variation. Each image corresponds to a different cutting speed: 1.5 m/min. Figure 9—a, 3 m/min Fig. 9—b, and 3.5 m/min Fig. 9 – c. These speeds represent increasing levels of energy interaction per unit time, which directly affects how clean or rough the surface appears under scanning electron microscopy.

In Fig. 9—a, where the cutting speed is 1.5 m/min, the SEM image reveals a surface characterized by deep, continuous vertical striations. These striations indicate unstable melt ejection behavior and poor fluidity of molten metal due to the prolonged laser interaction with the surface. The extended exposure time causes excessive heat input, resulting in increased surface roughness and the formation of macro-irregularities. Evidence of dross particles and partially re-solidified molten droplets is also visible on the surface, adhering to the striated walls. This signifies poor expulsion of molten metal and greater thermal damage, particularly in the initial phase of cutting. Overall, the surface appears rough and uneven, with distinct microstructural inconsistencies.

In contrast, Fig. 9—b, corresponding to a cutting speed of 3 m/min, shows a notable improvement in surface morphology. The striations remain visible but appear more evenly spaced and vertically aligned, indicating improved melt flow and better control over energy delivery. The reduction in heat input per unit length has minimized the size and depth of surface grooves. Furthermore, fewer particles and defects are observed on the surface, suggesting more effective melt ejection and less recast material remaining at the edges. As a result, the surface roughness is moderate, and the cutting start zone appears more uniform, representing a good balance between processing time and cut quality.

Figure 9 – c, arrested at the highest examined speed of 3.5 m/min, presents the smoothest surface among the three conditions. The SEM image shows faint or almost negligible striations, reflecting highly stable melt dynamics and minimized thermal influence. The reduced interaction time between the laser beam and the material allows for efficient material removal with minimal surface damage. However, some minor irregularities can still be seen, such as a spherical recast droplet and swirling traces near the lower boundary of the cut. These are likely artifacts of initial melt instability before the kerf fully develops. Despite these minor features, the surface appears clean, flat, and of high quality, indicating that higher cutting speeds, when properly controlled, lead to superior surface finishes and reduced post-processing requirements.

**Fig. 10:**
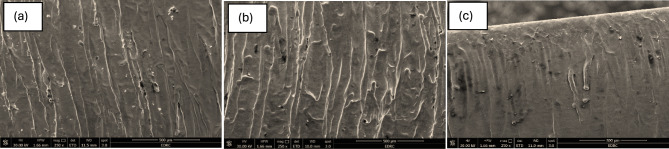
SEM images showing the mid cutting surface morphology of AISI 304 stainless steel under different laser cutting speeds: **a** Sample 1 at 1.5 m/min, **b** Sample 4 at 3.0 m/min, and **c** Sample 15 at 3.5 m/min.

The SEM micrographs shown in the attached Fig. 10 represent the mid-distance regions of laser-cut SS304 stainless steel samples at different cutting speeds: (a) 1.5 m/min, (b) 3.0 m/min, and (c) 3.5 m/min. These regions are critical for assessing the steady state cutting performance, as thermal stabilization typically occurs after the initial entry zone. The images help evaluate the influence of cutting speed on surface quality, roughness, melt flow consistency, and defect formation during continuous cutting.

In Fig. 10—a, captured at a cutting speed of 1.5 m/min, the surface exhibits dense and irregular striations, with notable roughness on the surface. The striations appear deeper and more erratic, indicating inconsistent melt removal and prolonged laser-material interaction. The thermal load at this slower speed results in excessive melting, leading to increased deposition of molten material that re-solidifies on the cut surface. Small cavities, droplets, and partially fused particles are observed, all of which contribute to a rougher and less uniform surface. This suggests that the melt ejection was inefficient, likely due to reduced gas dynamics and prolonged residence time of the beam over each unit length.

Figure 10 – b, corresponding to a speed of 3.0 m/min, reveals a much more organized and smoother cut surface. The striations are more parallel and evenly spaced, and the depth of the grooves appears reduced compared to the slower speed. The melt flow seems more controlled, with significantly fewer attached particles and smoother microstructural features. This improvement in surface quality can be attributed to a better balance between energy input and cutting speed, which helps maintain sufficient melting without overexposing the material. As a result, the surface roughness is moderate, and the kerf sidewalls are more homogeneous. The image reflects effective energy utilization and proper melt expulsion under optimized cutting conditions.

In Fig. 10 – c, where the cutting speed is increased to 3.5 m/min, the surface appears even smoother, with striations becoming faint and more dispersed. However, there are visible melt-related features such as scattered molten droplets and some recast material, which indicate a slightly unstable melt ejection process due to the reduced interaction time. Although overall surface roughness is the lowest among the three conditions, some localized irregularities may arise from insufficient melt removal or turbulence in the gas jet dynamics. This suggests that while higher cutting speeds improve cut smoothness and reduce heat-affected zones, they may also risk incomplete penetration or marginal quality consistency if not carefully controlled.

**Fig. 11:**
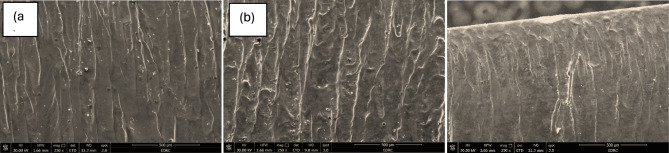
SEM images showing the end cutting surface morphology of AISI 304 stainless steel under different laser cutting speeds: **a** Sample 1 at 1.5 m/min, **b** Sample 4 at 3.0 m/min, and **c** Sample 15 at 3.5 m/min.

The SEM micrographs in the attached Fig. 11 depict the cut surface morphology at the end of the laser cutting path for SS304 stainless steel under three different cutting speeds: (a) 1.5 m/min, (b) 3.0 m/min, and (c) 3.5 m/min. The end of the cut is especially informative because it often reveals accumulated thermal effects, melt ejection stability, and overall consistency of the laser cutting process.

In Fig. 11—a, at the lowest cutting speed of 1.5 m/min, the surface exhibits pronounced and deeply etched striations that are closely packed and irregular in alignment. This indicates an unstable melt flow and poor melt ejection efficiency. As cutting progresses to the end, the extended exposure time and continuous heat input cause accumulation of thermal energy, which leads to an increase in surface roughness and the formation of recast layers and dross particles. The irregular striation pattern and clustered white specks suggest localized overheating, resolidified melt droplets, and possibly oxidation. Such surface morphology is typical of over-melting and poor energy balance, resulting in a degraded finish toward the end of the cut.

In Fig. 11 – b, at an intermediate cutting speed of 3.0 m/min, the cut surface becomes more uniform and refined. Striations are still visible but appear straighter, more evenly spaced, and shallower than in Fig. 11- a. This indicates improved melt dynamics and more stable energy delivery throughout the cutting process. While some micro-defects and small adhered particles are still present, they are less severe and more uniformly distributed. The roughness of the surface is moderate, reflecting effective thermal control and efficient melt removal. Importantly, the consistency of the striations from top to bottom of the field of view suggests that cutting stability was maintained throughout, even toward the termination of the path.

In Fig. 11 – c, at the highest cutting speed of 3.5 m/min, the cut surface is the smoothest among the three, with very fine and faint striations. The overall texture of the surface appears flatter and more homogeneous, which is indicative of minimal heat accumulation and efficient cutting with reduced interaction time. However, one can observe slight turbulence marks and fine droplets near the kerf edge, likely due to the laser beam’s reduced dwell time at the final point of the cut. These features may result from a small delay in melting expulsion at the cut exit, but they are relatively minor. The reduced roughness and smoother finish suggest that high-speed cutting enhances surface quality, particularly in the tail zone, although it must be balanced to avoid incomplete cuts or under penetration in thicker materials.

#### X-Ray diffraction (XRD) analysis of cut surface

According to the results of the surface characterizations and microhardness results of the tested samples, the Optimal Cutting Conditions for Key Performance Objectives in Fiber Laser Cutting of AISI 304 are determined as listed in Table 6. The balanced performance is achieved at the cutting conditions of 2 m/min cutting speed and 30% focus, as presented in Table 6 (Ra ≈ 4.1 µm, Kerf ≈ 140 µm, HV ≈ 270 HV). On the other hand, the worst combination of the cut edge properties is associated with 1.5 m/min cutting speed and 30% focus (Ra ≈ 4.8 µm, Kerf ≈ 142 µm, HV ≈ 119 HV). Based on these outcomes, samples of 1.5 m/min and 2 m/min cutting speed are subjected to additional metallography examinations. X- ray diffraction analysis had been carried out on the cut surface, as well as, HAZ was subjected to SEM, mapping, and EDS evaluation.

**Fig. 12:**
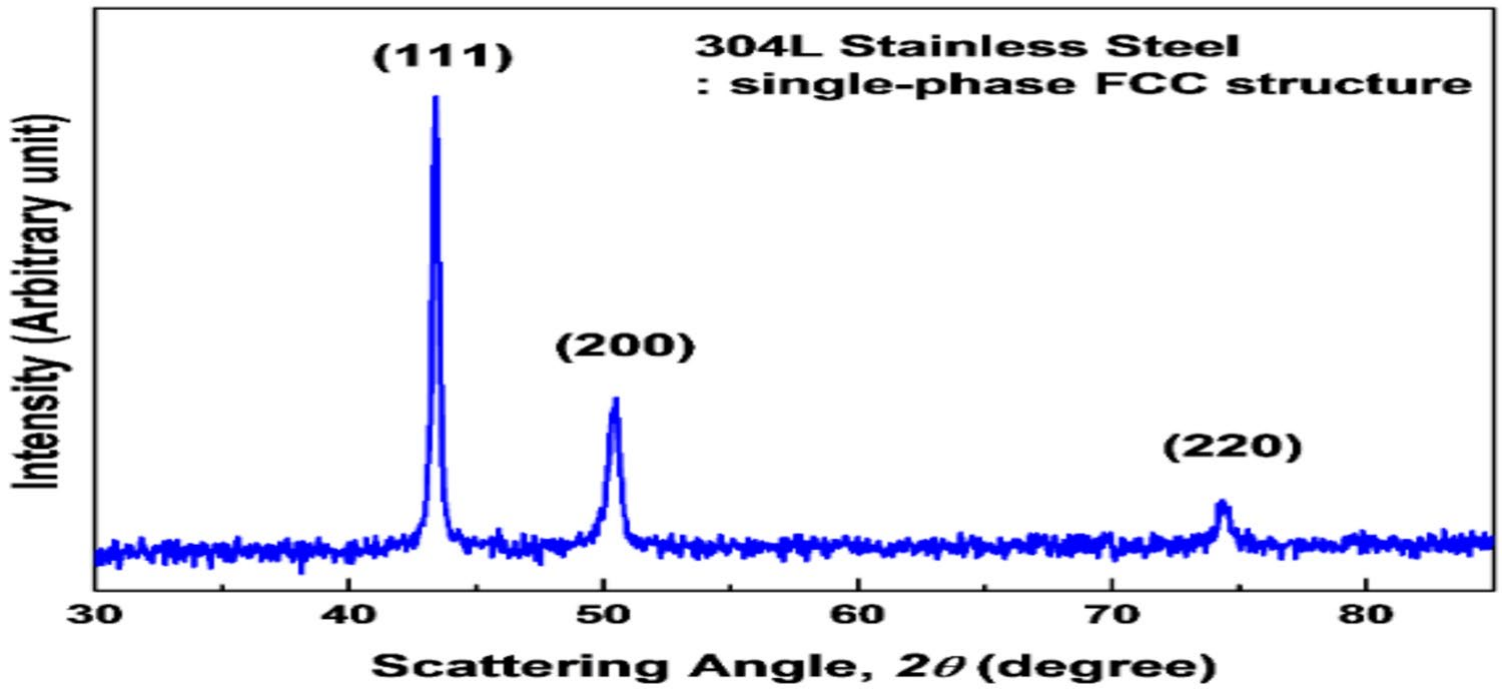
X-Ray diffraction pattern of AISI 304^49^.

**Fig. 13:**
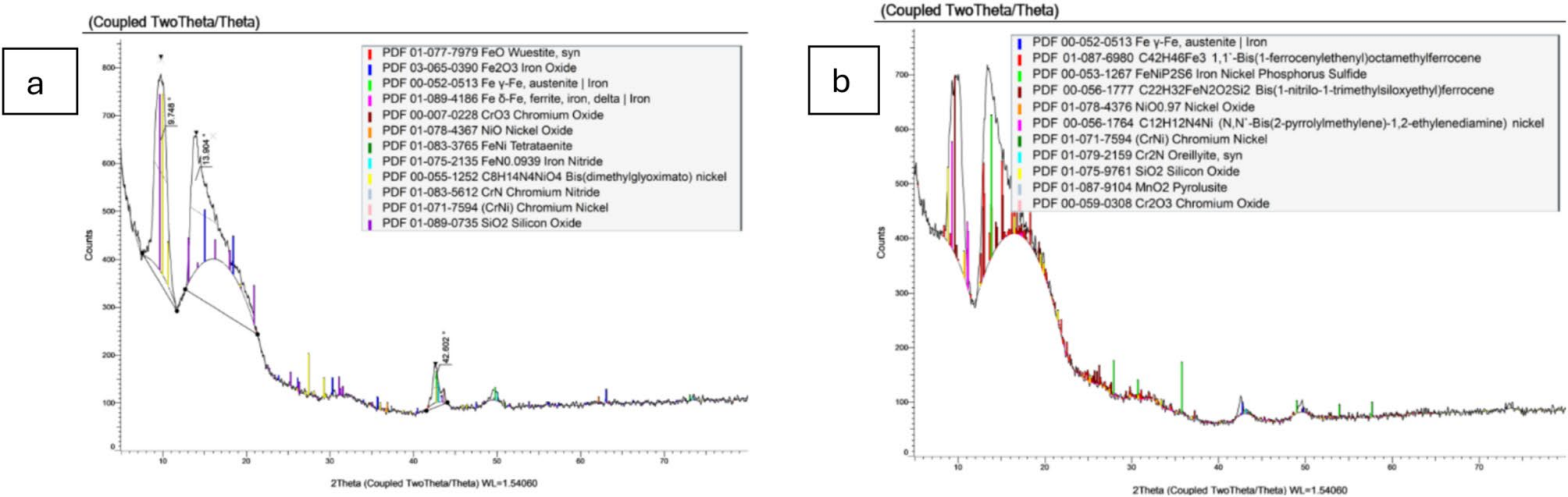
X-Ray diffraction pattern of tested samples at **a** 1.5 m/min, and **b** 2 m/min.

The single-phase face-centered cubic (FCC) structure of 304 stainless steel is depicted in the X-ray diffraction pattern in Fig. 12. In the current investigation, XRD-analysis of AISI 304, as displayed in Fig. 13, indicates that the metallography investigation shows a notable alteration in the cut edge’s microstructure. Tables 4 and 5 present the microstructural phase analysis for samples cut at speeds of 1.5 m/min and 2 m/min, respectively. Iron oxides identified in Fig. 13 include γ-Fe₂O₃ and δ-FeO, confirming the occurrence of oxidation under the lower cutting speed, whereas the sample cut at 2 m/min shows no evidence of iron oxide layer formation. This difference represents a notable and distinctive conclusion of the current study. On the other hand, intermetallic compounds were identified in both samples; however, the sample cut at 2 m/min speed exhibited the formation of beneficial intermetallic phases such as Cr₂O₃, NiO, CrNi, MnO₂, SiO₂, and Cr₂N. These compounds are hard particles, which contributes to enhanced hardness. Cr₂O₃, MnO₂ and Cr₂N play a particularly significant role in improving corrosion resistance. In contrast, the intermetallic compounds formed in the sample cut at 1.5 m/min cutting speed, namely NiO, CrNi, and SiO₂ are relatively hard but lack corrosion resistance, with CrN and CrO₃ representing thermodynamically unstable phases. Additionally, the cut edge of the sample produced at the lower cutting speed underwent a phase transformation from γ-Fe2O3 and δ-FeO,. Moreover, uncommon and complex intermetallic compounds such as C₈H₁₄N₄NiO₄, C₂₂H₃₂FeN₂OSi₂, etc., were also detected in both samples, indicating intricate chemical interactions during the cutting process.The deterioration in the microstructure is due to prolonged heat interaction at lower cutting speed- sample. Conversely, at higher speed, the heat interaction is reduced which promotes surface cleanliness from much oxidization layers and minimizes residual melt material on the cutting surface. This correlate with improved hardness and surface roughness. These outcomes interpret surface roughness and microhardness results (Fig. 6 & 8). Maciej Zubko et al., demonstrate oxidation formation in AISI 304 stainless steel cut surface by laser machine with Nitrogen gas asses^48^.

**Table 4:** X-Ray analysis of the 1.5 m/min cutting-speed sample.

Formula	Compound Name
FeO	Wüstite, syn
Fe₂O₃	Iron Oxide
γ-Fe	γ-Fe, austenite | Iron
δ-Fe	δ-Fe, ferrite, iron, delta | Iron
CrO₃	Chromium Oxide
NiO	Nickel Oxide
FeNi	Tetrataenite
FeN	Iron Nitride
C₈H₁₄N₄NiO₄	Bis(dimethylglyoximato) nickel
CrN	Chromium Nitride
CrNi	Chromium Nickel
SiO₂	Silicon Oxide

**Table 5:** X-Ray analysis of the 2 m/min cutting- speed sample.

Formula	Compound Name
γ-Fe	austenite Iron
C₄₂H₄₆Fe₃	Bis(1-ferrocenylethenyl)octamethylferrocene
FeNiP₂S₆	Iron Nickel Phosphorus Sulfide
C₂₂H₃₂FeN₂OSi₂	Bis(1-nitrilo-1-trimethylsiloxyethyl)ferrocene
NiO	Nickel Oxide
C₁₂H₁₂N₄Ni	(N,N`-Bis(2-pyrrolylmethylene)-1,2-ethylenediamine)
CrNi	Chromium Nickel
Cr₂N	Chromium nitride)
SiO₂	Silicon Oxide
MnO₂	Manganese dioxide
Cr₂O₃	Chromium Oxide

Finally, XRD evaluation of the cut surface indicates that the cutting speed should not be too low, as this may lead to excessive thermal interaction and surface deterioration. Conversely, it should not be too high, in order to allow sufficient time for favorable surface precipitation, which can enhance the material’s properties.

#### SEM, Mapping, and EDS Evaluation of HAZ

**Fig. 14:**
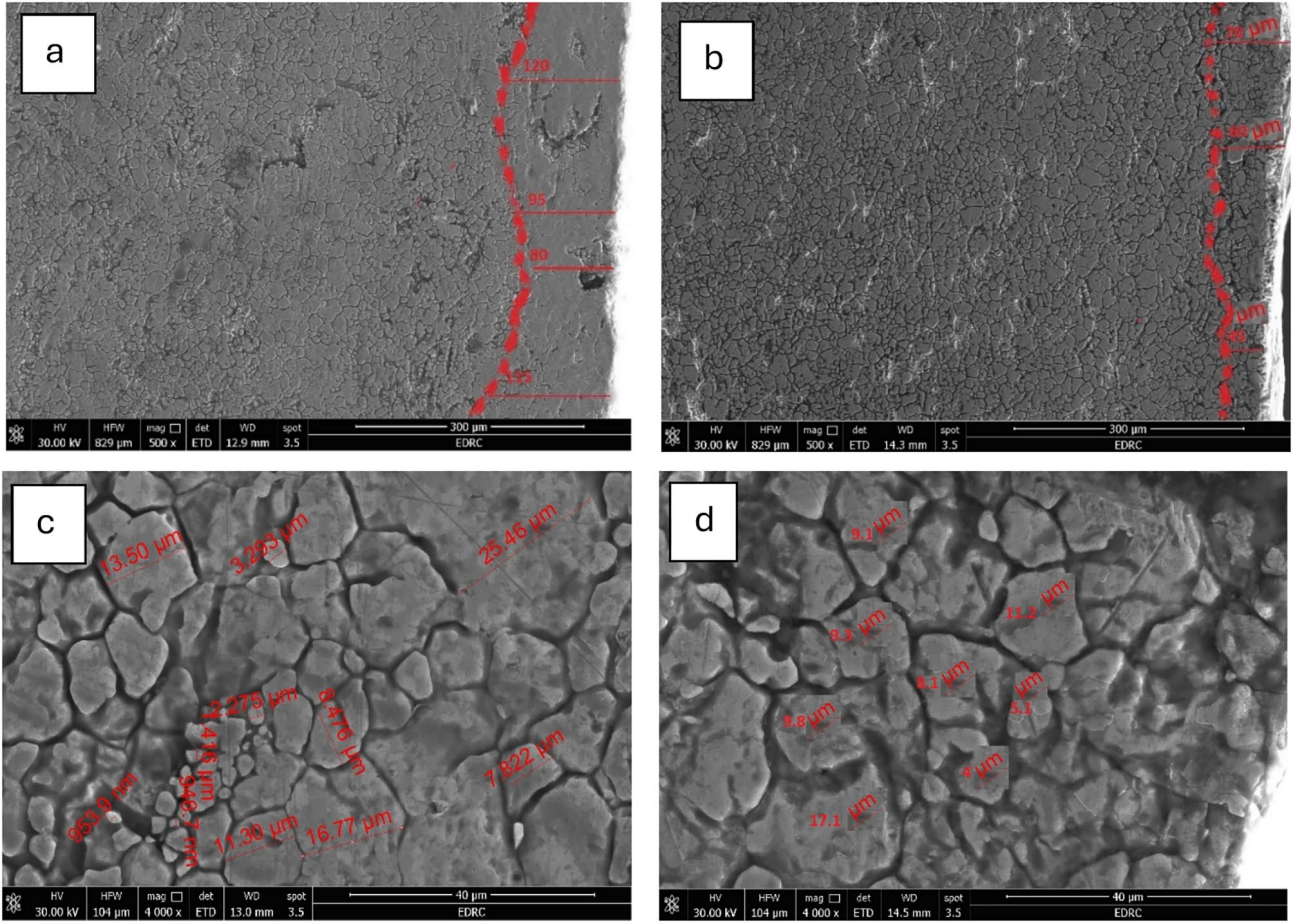
SEM images of: **a** HAZ width of the 1.5 m/min cutting-speed sample, **b** HAZ width of the 2 m/min cutting-speed sample, **c** Grain size of the 1.5 m/min cutting-speed sample, and **d** Grain size of the 2 m/min cuttin-speed sample.

**Fig. 15:**
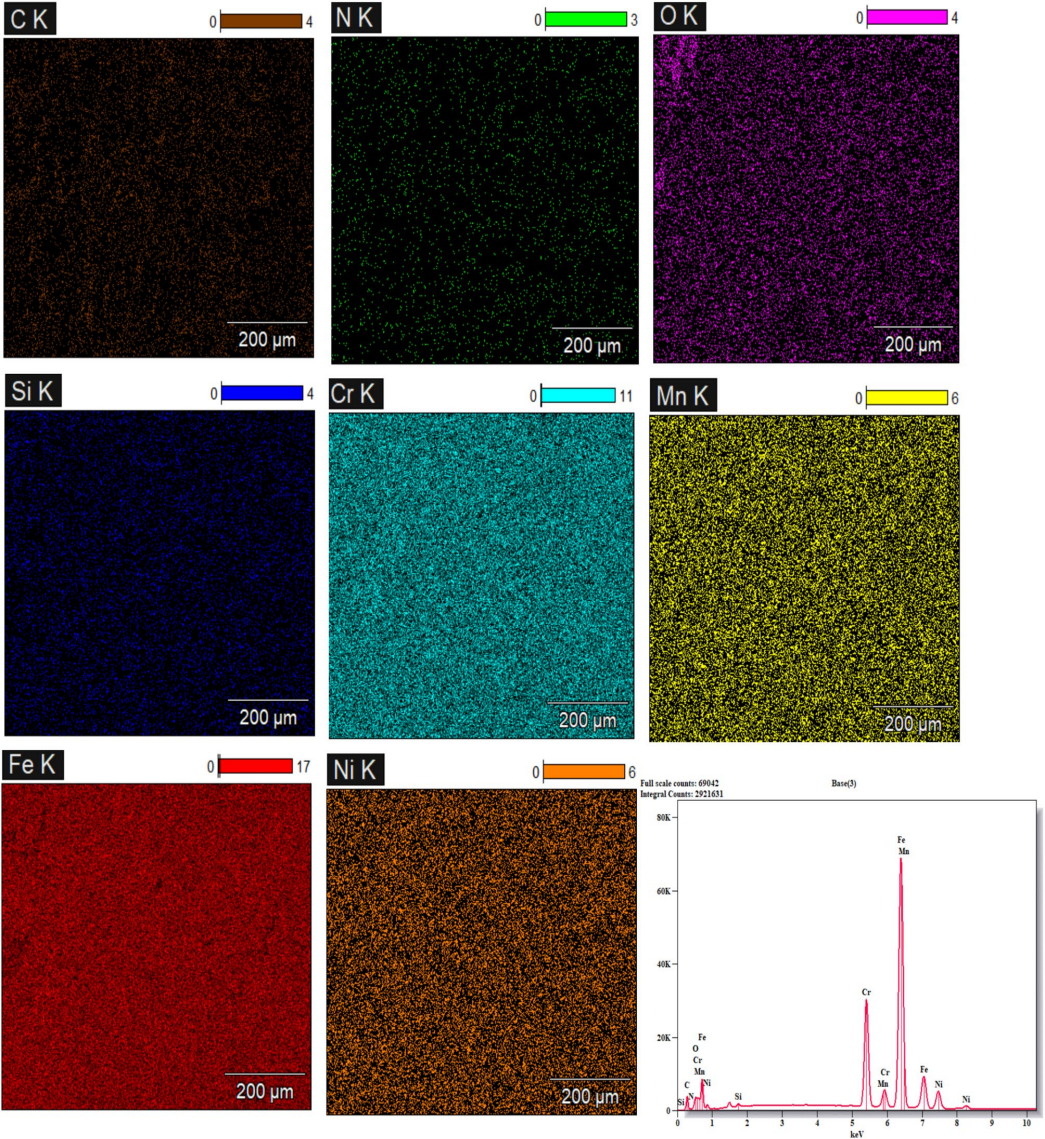
Mapping and EDS analyses of the 1.5 m/min cutting-speed sample.

**Fig. 16:**
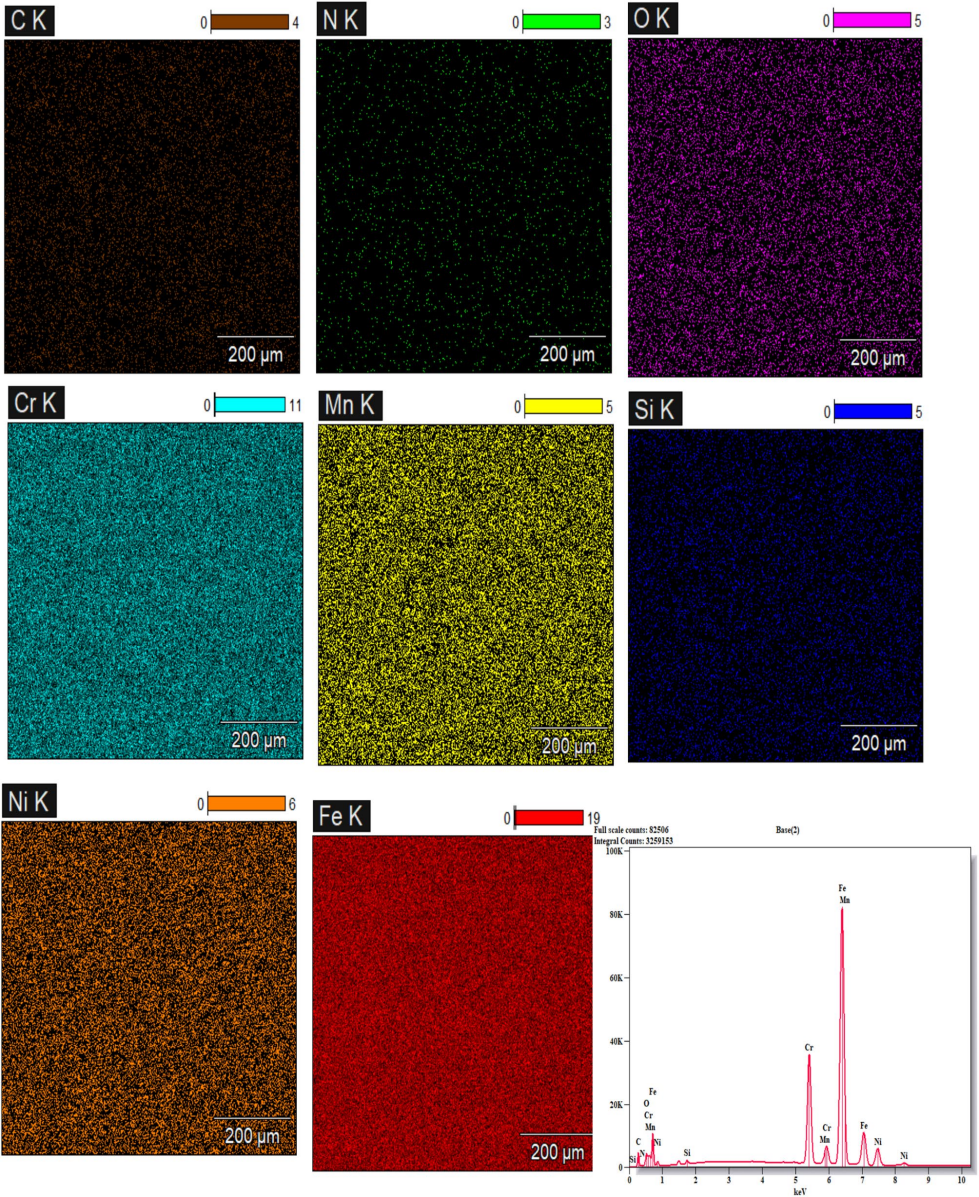
Mapping and EDS analyses of the 2 m/min cutting-speed sample.

The results of SEM, mapping, and EDS analysis Figs. 14, 15, and 16, respectively. As presented in Fig. 14, by increasing the cutting speed from 1.5 m/min to 2 m/min, the HAZ average width is reduced from 102 µm to 56.7 µm. The larger heat-affected zone (HAZ) observed at low cutting speeds is attributed to the higher thermal energy input per unit area, which also leads to increased molten metal deposition on the cut surface, resulting in higher surface roughness of the laser-cut edge.

This outcome is consistent with the surface roughness measurements in the current study. Whereas, by increasing the cutting speed from 1.5 m/min to 2 m/min, the surface roughness is reduced from 4.8 µm to 4.1 µm, as illustrated previously in Fig. 6. This primarily attributed the reduced thermal energy at higher cutting speeds. Previous studies have shown a similar trend, according to a study by B S Wardhana et al. on laser-cut 316L stainless steel, the extent of the heat-affected zone (HAZ) contributes to increased residual melt material on the cut surface. Consequently, the surface roughness of the laser-cut edge increased^36^. A study by Chandan and GK et al. Demonstrated that lowering the average laser power can assist reduce the area of the heat-affected zone while keeping other parameters constant^50,51^. L Łatka et al., examined laser-cut S355J2 structural steel, they found that the HAZ decreased as the cutting speed increased^52^.

Additionally, as shown in Fig. 14-a, microcracks were observed in HAZ of lower cutting-speed Sample, which also exhibited pronounced grain coarsening. The observed grain growth is attributed to the prolonged thermal exposure associated with the lower cutting speed, while the formation of microcracks is likely a consequence of an inadequate thermal cycle during the cutting process.

On the other hand, mapping and EDS analyses were performed on the heat-affected zones (HAZ) of the same samples, as illustrated in Figs. 15 and 16, respectively. The results reveal notable alterations in the microstructural phases compared to the standard microstructure (Fig. 12). The presence of oxygen (O) detected in the EDS spectra of the examined regions indicates the formation of oxide compounds, irrespective of whether their effect on the material properties is beneficial or detrimental. However, as discussed above in XRD section, all oxides formed in the cut edge of higher cutting speed (2 m/min) are beneficial such as Cr₂O₃, NiO, MnO₂, SiO₂,) while most of detected oxides at the cut surface of lower cutting speed sample are detrimental like; Fe2O3, FeO, CrO₃.

Furthermore, the presence of Nitrogen (N) signals possible interaction between the metal surface and the shielding nitrogen gas during the cutting process. Nevertheless, as previously discussed, a beneficial compound like Cr₂N has been formed at the edge when cut at a speed of (2 m/min), whereas an unstable thermodynamic phase such as CrN appeared on the cut surface at a cutting speed of (1.5 m/min).

Ultimately, the findings form metallographic analyses of HAZ, and cut edges, support the experimental results of the current investigation, including those related to surface roughness, kerf width, and non-linear behavior of microhardness.

### Optimal cutting parameters

In the current study, a comprehensive experimental investigation has been conducted to assess the interactions between laser parameters and the thermo-mechanical response of 4 mm-thick AISI 304 stainless-steel. The main objective is to develop optimization frameworks that minimize energy consumption while maintaining cutting precision and material performance, thereby contributing to the advancement of next-generation sustainable manufacturing technologies. Table 6 summarizes optimal cutting parameters for AISI 304 stainless steel, highlighting the best conditions for surface roughness, kerf width, microhardness, and a well-balanced performance in all parameters. The minimum surface roughness (Ra = 3.97 µm) was achieved at a cutting speed of 2.5 m/min with a focus position of 30%. This condition reflects a balanced heat input and interaction time at a moderate cutting speed. In contrast, both excessively low and high speeds lead to increased roughness due to, respectively, excessive melting or incomplete cutting. The narrowest kerf width (≈138 µm) was obtained at a cutting speed of 3.5 m/min and 30% focus. In this condition, the reduced energy per unit length limits heat diffusion, thereby confining the kerf and improving dimensional accuracy. The maximum microhardness (≈270 HV) was recorded at the cutting speed of 2 m/min with a 30% focus, where higher energy absorption promotes rapid solidification and grain refinement. While this increases hardness, it also tends to widen the kerf and slightly deteriorate the surface finish. A balanced performance (HV ≈ 270 HV, Ra ≈ 4.1 µm, Kerf ≈ 140 µm) was observed at 2 m/min and 30% focus, providing a practical compromise between hardness, kerf width, and surface roughness. This condition represents the most suitable setting for reliable and consistent processing of AISI 304 fiber laser cutting. Considering cost-effectiveness, a moderate cutting speed combined with a medium assist gas pressure (14 bar) produced a high-quality cut with balanced performance. Moreover, the moderate consumption of both power and gas, together with the advantages of material and gas costs discussed in the introduction, further supports the suitability of this parameter range.

**Table 6:** Optimal cutting conditions for key performance objectives in fiber laser cutting of AISI 304.

Objective	Cutting Speed (m/min)	Focus Position (%)	Result
Minimum Surface Roughness (Ra)	2.5	30	Ra ≈ 3.97 µm (smoothest surface)
Narrowest Kerf Width	3.5	30	Kerf ≈ 138 µm
Maximum Microhardness	2	30	Hardness ≈ 270 HV
An overall balanced performance of all parameters	2	30	Ra ≈ 4.1 µm, Kerf ≈ 140 µm, HV ≈ 270 HV

## Conclusions

This study comprehensively examined the effects of cutting speed and focus concentration on the surface integrity, kerf geometry, and hardness of AISI 304 stainless steel using a 2000W fiber laser cutting system. The following conclusions can be drawn:

- Fifteen experimental trials were conducted, with cutting speeds ranging from 1.5 to 3.5 m/min and focus concentration ratios ranging from 30 to 50%, while maintaining constant values ​​for laser power of 200 W (100%) and gas pressure (14 bar). The results indicate a strong correlation between laser cutting parameters and the resulting surface properties.
- Surface roughness (Ra) is highly sensitive to changes in cutting speed. At the lowest speed (1.5 m/min), Ra values ​​peaked, reaching 5.4 µm at 40% focus. This is attributed to prolonged laser-material interaction, which resulted in increased heat input and reduced melting ejection. As cutting speed increased, Ra values ​​consistently decreased. At 2.5 m/min and 30% concentration, the lowest Ra recorded was 3.97 µm, indicating that higher speeds lead to cleaner cuts by reducing energy density per unit length. Concentration position also affected roughness, with 30% consistently producing smoother surfaces at most cutting speeds. For example, at 3.5 m/min, Ra decreased from 4.2 to 4.0 µm for focus change from 40 to 30% respectively.
- Kerf width showed a parallel trend. At 1.5 m/min and 50% concentration, the kerf width reached its widest at 185 µm, while reducing the concentration to 30% at the same cutting speed narrowed the kerf to 138 µm (25% improvement). This decrease indicates that lower focus percentages concentrate the laser energy more effectively, resulting in more precise material removal and crack narrowing.
- The non-linear hardness behavior within such a narrow speed range of 1.5–3.5 m/min is a distinctive finding in this study. At 2.0 m/min and 30% concentration, the highest hardness value of 270 HV was recorded, indicating that this condition promoted optimal thermal gradients and cooling rates for microstructure improvement, as expected. Conversely, at 1.5 m/min and 30% concentration, the hardness decreased to approximately 115 HV, reflecting thermal softening due to extended heating. Similar behavior was observed at cutting speed of 3.5 m/min and a concentration of 30%, the hardness decreased further to approximately 145 V Hz, likely due to insufficient thermal energy to induce structural hardening. Therefore, the optimal range for microhardness enhancement lies between a cutting speed of 2.0 and 2.5 m/min with concentration settings of 30% and 50%.
- Cutting speed and focus concentration optimization is essential to achieve balance between cut quality, precision, and mechanical performance. The most favorable combination was observed at 2 m/min with a 30% focus, resulting in the moderate surface roughness (Ra ≈ 4.1 µm), an acceptable kerf width (~ 140 µm), and high microhardness (~ 270 HV). For applications requiring high precision, and moderate hardness, settings around 2.5 m/min and a 30% focus provide results of (Ra ≈ 3.8 µm and ~ 195 HV). These results provide practical and sustainable guidelines for precision machining using fiber laser cutting in stainless steel processing.
- Scanning electron microscope (SEM) evaluations at the start, middle, and end of the cut confirmed the quantitative results. At 1.5 m/min, surfaces in all regions exhibit deep striations, adherent melt droplets, and an inhomogeneous texture, particularly at the end of the cut, indicating unstable melt dynamics and heat buildup. At 3.0 m/min, SEM images showed straighter striations, fewer re-solidified particles, and smoother kerf walls, supporting the measured improvements in Ra and kerf width. The highest cutting speed, 3.5 m/min, yielded smoother, flatter surfaces with minimal visible defects and consistent shape, although minor signs of turbulence and melt vortex were observed at the end of the cut.
- Additional metallographic examinations, including SEM, mapping, and EDS analyses, were conducted on selected samples to interpret the contrasting results between the balanced performance observed at a cutting speed of 2 m/min and the poor performance associated with the 1.5 m/min cutting speed, both carried out with the same laser focus (30%). XRD techniques were also employed to support the analysis. The SEM imaging results indicate a thinner heat-affected zone (HAZ) at a cutting speed of 2 m/min, whereas samples cut at the lower speed of 1.5 m/min exhibit significant grain coarsening. Mapping and EDS analyses further reveal notable changes in the microstructural phases within the HAZ of the cut samples. The detection of oxygen (O) in the and mapping and EDS spectra suggests the formation of oxide compounds in the HAZ. XRD analysis of the cut surface of 1.5 m/min, and 2 m/min cutting-speed samples evidences various phases formation. The oxides formed at the cut surface of higher cutting-speed sample are favorable such as Cr₂O₃, NiO, MnO₂, SiO₂,) while most of detected oxides at the cut edge of lower cutting-speed sample are detrimental like Fe2O3, FeO, and CrO₃.
- Ultimately, the metallographic analysis findings of the HAZ and cut edges reinforce the experimental results of the current investigation, particularly those concerning surface roughness, kerf width, and non-linear behavior of microhardness.

## Future work

For advancing laser cutting process optimization and enhancing industrial competitiveness, there is a pressing need for in-depth experimental and metallographic investigations to elucidate the complex interactions between laser processing parameters and the thermo-mechanical response of AISI 304 stainless steel.

## Data Availability

The datasets used and/or analyzed during the current study available from the corresponding author on reasonable request.

## Abbreviations

AISI: American iron and steel institute
SS304: Stainless steel grade 304
Ra: Arithmetical mean surface roughness
Rz: Average distance between highest peak and lowest valley
SEM: Scanning electron microscopy
FESEM: Field emission scanning electron microscopy
LSCM: Laser scanning confocal microscopy
HV: Vickers hardness (Hardness Value)
HAZ: Heat-affected zone
SOD: Stand-off distance
IPG: (Manufacturer name for fiber laser, e.g., IPG Photonics)
TR 200: Surface roughness tester (Model)
CYPCUT: Laser control software (Shanghai Bochu)
